# Phase Composition, Microstructure, Multiple Shape Memory Effect of TiNi_50−x_V_x_ (x = 1; 2; 4 at.%) System Alloys

**DOI:** 10.3390/ma15238359

**Published:** 2022-11-24

**Authors:** Ekaterina Marchenko, Alexander Monogenov, Anatoly Klopotov, Gulsharat Baigonakova, Ekaterina Chudinova, Alexander Vorozhtsov, Sergei Sokolov

**Affiliations:** Laboratory of Superelastic Biointerfaces, National Research Tomsk State University, 36 Lenin Ave., 634045 Tomsk, Russia

**Keywords:** titanium nickelide, alloying, shape memory effect, vanadium, martensitic transformation, microstructure

## Abstract

The phase composition, microstructure, and multiple shape memory effect of TiNi_50−x_V_x_ alloys were studied in this work. The phase composition of the TiNi_50−x_V_x_ system is the TiNi matrix, spherical particles of TiNiV, the secondary phase Ti_2_Ni(V). Doping of TiNi alloys with vanadium atoms leads to an increase in the stability of high-temperature B2 and rhombohedral R-phases. An increase in the atomic volume with an increase in the concentration of the alloying element V from 1 to 4 at.% was established. Vanadium doping of the Ti–Ni–V system alloys leads to an increase in the temperature interval for the manifestation of the multiple shape memory effect. It has been established that the value of the reversible deformation of the multiple shape memory effect both during heating and during cooling increases linearly from 2 to 4% with an increase in the vanadium concentration.

## 1. Introduction

Alloys based on titanium nickelide are used in medicine, aviation or automotive industry as supporting, executive or fixing elements [1,2,3,4,5]. High rate of shape memory properties and superelasticity of certain alloy compositions based on titanium nickelide, as well as high corrosion resistance are an indisputable advantage of titanium nickelide alloys. In the last decade, significant efforts have been made to modify the chemical composition of NiTi-based alloys (by ternary and quaternary substitution of Ni and/or Ti) to increase the transformation temperature [6], control hysteresis [7], and/or improve mechanical properties such as strength and corrosion resistance [8]. Much attention is paid to the question of what position of the titanium or nickel atoms will be occupied by one or another alloying element; however, there is not enough clarity in this issue [9,10,11].

The doping of equiatomic alloys with vanadium remains poorly covered in the literature. It is noted that with an increased content of nickel, vanadium replaces titanium, and with an increased content of titanium, vanadium occupies the nickel sublattice [9]. The physical and mechanical properties of the Ti–Ni–V system will differ greatly depending on which of the main components is enriched in the TiNi alloy.

There are a number of publications where composites based on titanium nickelide and nanosized vanadium filaments have been studied. In this case, the superelastic properties of titanium nickelide alloys were improved with thin vanadium nanowires [12]. It is known that doping with vanadium strengthens the alloys; as a negative effect, the formation of particles of the second phase (Ti,V)_2_Ni in the NiTi-V matrix absorbs oxygen atoms with the formation of the oxide (Ti,V)_4_Ni_2_O, which, in turn, worsens the behavior of the alloys with shape memory with increasing vanadium content [13]. It is noted that there is an expansion of the temperature range of direct and reverse martensitic transformation (MT), an increase in the martensitic shear stress and the magnitude of reversible deformation with the shape memory effect with significant alloying V up to 25 at.%, instead of Ti and Ni [14]. In another work, an alloy wire with a V content of 40 at.% was obtained by casting, forging and drawing. The characteristics of MT wires made of the V_40_Ni_31_Ti_29_ alloy have been studied. The results show that the critical temperatures of the B19’ → B2 MT exceed 100 °C. The MT temperature range is about 80 °C, which is much higher than that of NiTiV alloys with a lower content of the vanadium [15]. There are reports that doping with tungsten up to 4 at.% reduces the phase transition temperature of NiTi alloys, which leads to an improvement in the shape memory effect and an increase in superelasticity [16,17]. The effect of heat treatment on the phase transformation temperatures of NiTiV alloys was analyzed. The results showed that the addition of vanadium can promote the formation of an intermediate R-phase and affect the grain size and electrical resistivity [18]. According to studies of the phase transformation characteristics and behavior during isothermal oxidation of NiTiV alloys, it was found that the vanadium content can strongly affect the microstructure of the oxide film [19]. The influence of V on the evolution of the microstructure and mechanical properties of alloys enriched in nickel (60NiTi) was elucidated in [20]. The alloy consists of the NiTi matrix, Ni_4_Ti_3_ precipitates, and a small amount of the Ti_2_Ni phase. When the content of V is more than 3 at.%, alloys are coated with a Ni_3_Ti_2_ network structure, and when the vanadium content was raised to 10 at.%, the network structure of the Ni_3_Ti_2_ partially transferred to the Ni_3_Ti phase. In addition, the morphology and distribution of the Ni_3_Ti_2_ phase changed with increasing vanadium content. It was noted that the hardness of the alloys increased with increasing V content, while the fracture toughness and tensile strength first increased and then decreased due to the effect of the solid solution strengthening with vanadium and strengthening the Ni_3_Ti_2_ phase. It is shown that the addition of 3 at.% V was the most effective for improving the microstructure and properties of alloys highly enriched in nickel. Adding less than 3 at.% V improved the oxidation resistance at 500 °C.

A literature review of TiNiV alloys showed that alloying with vanadium is interesting not only from a scientific point of view but is also promising for solving the problem of increasing the amount of accumulated deformation with a multiple shape memory effect (SME) and strength properties. The aim of this work is to study the effect of vanadium concentration on the phase composition, structure, shape memory parameters and to analyze the change in the crystal structure during the dissolution of the alloying element in the matrix phase. Doping with vanadium up to 4 at.% instead of a part of nickel in alloys close to the equiatomic composition are investigated. In this work, for the first time, a deeper investigation of the structure of the Ti–Ni–V system is presented, namely, an analysis of the change in the crystal structure is carried out, concentration dependences of atomic volumes in binary and three-component systems Ti–V, Ni–V, Ni–Ti and Ti_50_Ni_50−X_V_X_ were obtained, the change in the atomic volume in the R-phase in alloys with different concentrations of vanadium was calculated to estimate the dissolution of the alloying element in the matrix phase. An experimental study of the multiple shape memory effect of TiNi-based alloys doped with V during thermal cycling was also carried out.

## 2. Materials and Methods

Ti–Ni–V system alloys were fabricated in an ISV-0.004 PI M1 induction furnace by remelting spongy titanium and N1 grade nickel. High purity vanadium (>99.99 wt.%) was used as a dopant. In the work, alloys based on TiNi of the following compositions Ti_50_Ni_49_V_1_; Ti_50_Ni_48_V_2_; Ti_50_Ni_46_V_4_ are named as alloy No. 1, alloy No. 2, alloy No. 3, respectively. The obtained ingots with a length of 15 cm and a diameter of 2 cm were rolled into strips, from which samples with dimensions of 50 × 1 × 1 mm^3^ were cut on an electro-erosive machine A 20786 to study mechanical properties. For structural studies, plates 10 × 1.5 × 1.5 mm^3^ in size were cut out. X-ray diffraction studies of the samples were carried out on a Shimadzu XRD-6000 diffractometer using Cu Kα radiation (1.5410 Å wavelength) and Co Kα (1.7890 Å wavelength). The diffraction patterns were indexed using the PowderCell 2.4 program. The microstructure of the alloys was studied using a Carl Zeiss Axiovert 40 MAT optical microscope. Elemental microanalysis of the matrix and phase precipitates was examined by scanning electron microscopy in a PHILIPS SEM 515 SEM using an EDAX ECON IV microanalyzer. To study the microstructure, thin sections were prepared in a standard way. To reveal the microstructure, a solution of hydrofluoric and nitric acids was used (3 mL HF, 2 mL HNO_3_, 95 mL H_2_O). Multiple SME with a constant external load was carried out by measuring macrostrain during thermal cycling under tension loading on an Instron 3369 universal testing machine with a cooling–heating chamber. 

## 3. Results

### 3.1. Phase Composition of Alloys Based on Titanium Nickelide Doped with Vanadium

**Figure 1:**
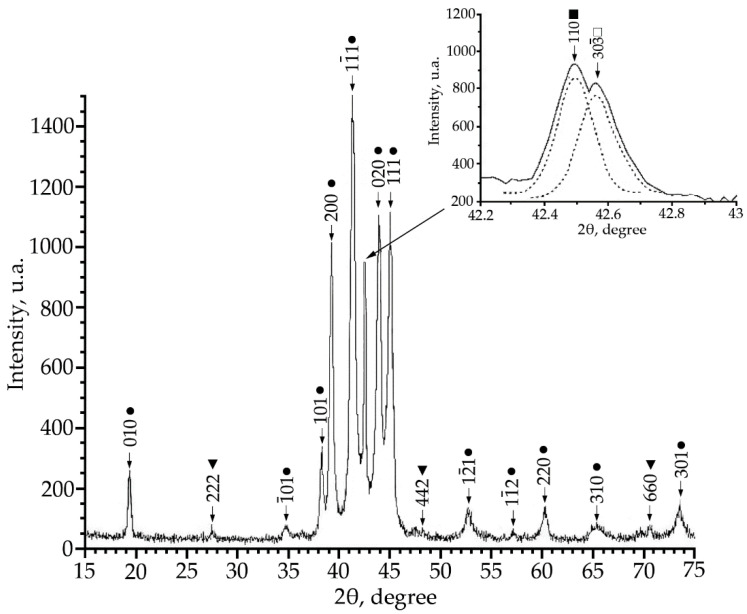
X-ray patterns (Cu Kα radiation) from alloy No. 1, where: •—martensitic phase B19′; ■—B2-phase; ▼—Ti_2_Ni secondary phase; □—R-phase.

**Figure 2:**
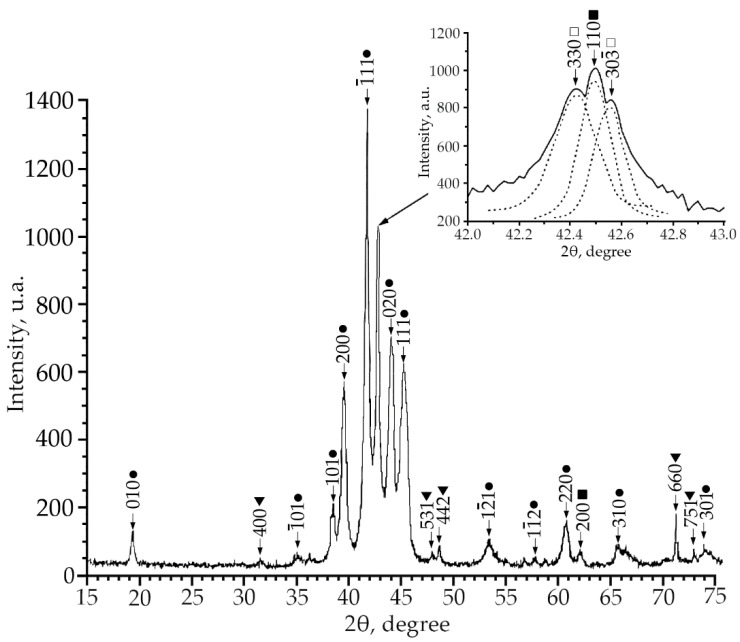
X-ray patterns (Cu Kα radiation) from alloy No. 2, where: •—martensitic phase B19′; ■—B2-phase; ▼—Ti_2_Ni secondary phase; □—R-phase.

**Figure 3:**
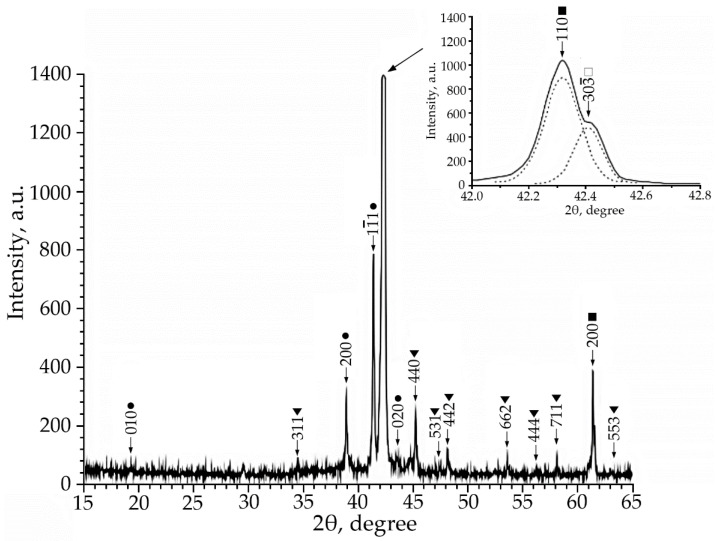
X-ray patterns (Co Kα radiation) from alloy No. 3, where: •—martensitic phase B19′; ■—B2-phase; ▼—Ti_2_Ni secondary phase; □—R-phase.

Using X-ray phase analysis, it was found that in alloys No. 1–3 at room temperature there is a multiphase mixture: an intermetallic compound based on titanium nickelide TiNi(V), which is in three crystallographic modifications (B2, R and B19′-structures) and the Ti_2_Ni compound (V) (Figure 1, Figure 2 and Figure 3).

The alloy No. 1 (V = 1 at.%) showed a large content of the low-temperature phase B19′ (Figure 1), this is evidenced by the angular distribution and the high intensity of reflections of this phase. An analysis of the reflection profile in the region of angles 2θ = 42 ÷ 43° revealed that the reflection consists of two doublets. The interpretation of these doublets made it possible to establish that there is a superposition of reflections from the B2 phase and the R phase (inset in Figure 1). The presence of structural lines of the Ti_2_Ni secondary phase was also found, and the volume fraction of the Ti_2_Ni secondary phase does not exceed 5%. 

According to the X-ray diffraction patterns of alloy No. 2 (V = 2 at.%) and alloy No. 1, structural reflections from the B19′ martensite phase are observed with high intensity. A triplet was found in the region of the (110)B2 structural reflection. The reflections of the R-phase (lines (330) and (30$\bar{3}$) and the reflection from the initial phase B2 (reflection (110)) of the triplet were determined (Figure 2). Analysis of the X-ray diffraction pattern of alloy No. 3 (V = 4 at.%) (Figure 3) revealed that the main phase at room temperature is the R phase. This is confirmed by the ratio of the intensities of reflections from the B2, B19′, Ti_2_Ni, and R phases on the X-ray diffraction pattern. In addition, the X-ray diffraction pattern of this alloy revealed a significant content of reflections from the secondary phase of the Ti_2_Ni type (Figure 3). The intensity of reflections of the martensitic phase with B19′ structure is low, which indicates a small amount of this phase. The described X-ray diffraction patterns clearly show that the volume fraction of the Ti_2_Ni phase is practically independent of the concentration V.

**Figure 4:**
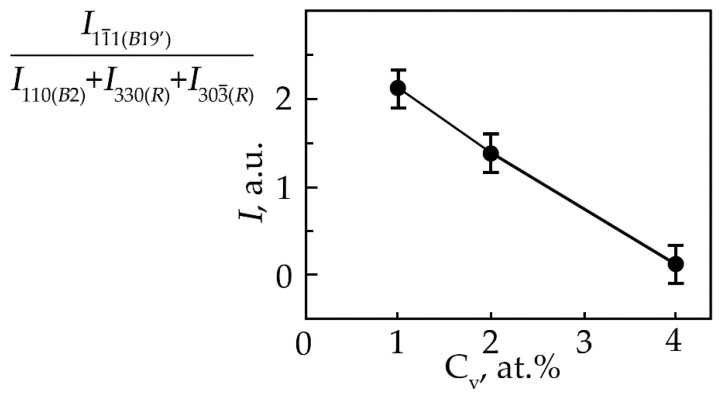
Dependence of the ratio of reflection intensities ($1\bar{1}1$) from the martensitic phase with the B19′ structure to the total intensity of the (330) and (30$\bar{3}$) R-phase reflections, which form a doublet upon splitting of the (110) reflection of the B2 phase at room temperature in TiNi alloys doped with vanadium, on the concentration V.

It has been established that the concentration dependence of the ratio of reflection intensities ($1\bar{1}1$) from the phase with the B19′ structure to the total intensity of the (330) and (30$\bar{3}$) R-phase reflections, which form a doublet upon splitting of the (110) reflection of the B2 phase (Figure 4), decreases monotonically from an increase in the concentration of V atoms.

This indicates that the volume fraction of the B19′ phase at room temperature decreases with an increase in the concentration of V atoms. Such a trend of the change in the structural-phase composition indicates that doping of TiNi alloys with vanadium atoms leads to an increase in the high-temperature stability of *B2* and the rhombohedral R-phase. It should be noted that the ratio of the intensities of the doublet lines of the R-phase is different (Figure 1, Figure 2 and Figure 3). In alloys with a minimum concentration of V atoms (1 at.%), the intensity of the reflection *I* (330)*_R_* exceeds *I* (30$\bar{3}$)*_R_*, at 2 at.% V, the intensities of the doublet lines are almost equalized; an increase in the concentration of V to 4 at.% leads to an increase in the intensity of the reflection *I* (330)*_R_* over *I* (30$\bar{3}$)*_R_* by approximately two times. The reason for the asymmetry in the intensity of the (330) and (30$\bar{3}$) doublet reflections is described in [21]. This distribution of reflection intensities can be due to various reasons. The first is the superposition of a single line of the residual phase B2 on the doublet of the R phase; the second is the presence of the R-phase texture inherited from the B2 phase or acquired during the B2→R phase transition.

**Figure 5:**
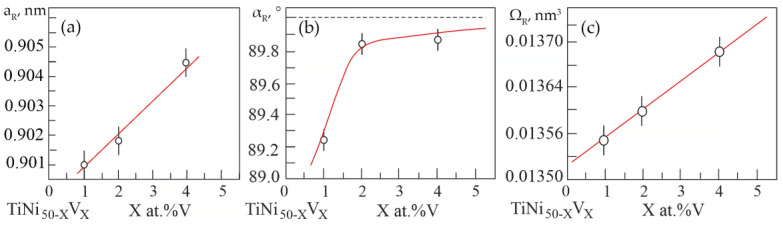
Concentration dependences: (**a**)—lattice parameter; (**b**)—rhombohedricity angle; (**c**)—atomic volume in the R-phase of alloys based on TiNi doped with V.

Based on the results of X-ray diffraction analysis, the dependences of changes in the unit cell parameters and atomic volume in the R-phase on the concentration V were determined (Figure 5).

It can be seen that an increase in the concentration V leads to an increase not only in the unit cell parameter of the R phase (Figure 5a), but also in the rhombohedricity angle αR (Figure 5b). This indicates an increase in distortions in the crystal lattice of the R-phase relative to the B2 cubic lattice. The linear unit cell parameter increases as the unit cell angular parameter α tends to the value of 90° in compounds based on TiNi with R-phase. Such a correlated change in the linear parameter of the unit cell by the angular parameter α in the R-phase in TiNi-based alloys is associated with a change in the forces of interatomic interaction. These interatomic changes are well manifested in the behavior of the of the elastic modulus of TiNi-based alloys. In order to analyze the change in the crystal structure and evaluate the dissolution of the doping element in the matrix phase, it was calculated the change in the atomic volume in the R-phase in alloys with different concentrations of vanadium (Figure 5c). An increase in the atomic volume with an increase in the concentration of the alloying element has been established.

### 3.2. Microstructure of TiNi-Based Alloys Alloyed with V

**Figure 6:**
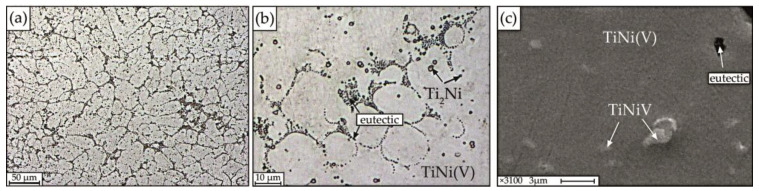
Microstructure of alloy No.1 obtained on optical (**a**,**b**) and scanning electron microscopes (**c**).

**Figure 7:**
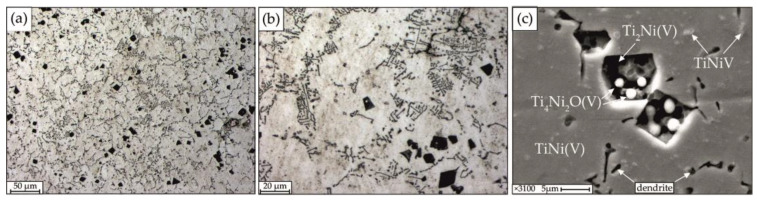
Microstructure of alloy No. 2 obtained on optical (**a**,**b**) and scanning electron microscopes (**c**).

The results of microstructural studies of thin sections of alloys No. 1–3 are presented in Figure 6 and Figure 7. The analysis of micrographs shows that the microstructure of the studied alloys varies with the concentration V.

The microstructure of the alloy with 1 at.% V is chemically and structurally inhomogeneous, which is clearly manifested in the form of regions of different morphology with pronounced dendritic cell borders, between which the eutectic (interdendritic eutectic) crystallizes, individual small particles of regular rounded shape, ranging in size from 1 to 2.5 μm and areas of solid solution (Figure 6). 

In addition, it can be seen that the morphology of the dendrites is different. Figure 6a, demonstrates a photograph showing a part of a columnar dendrite, in which the first order axis is directed perpendicular to the plane of the figure, and the secondary branches (second order axes) are elongated in different directions. According to local microanalysis, the following structure was identified: matrix based on B2 intermetallic compound—TiNi(V); fine particles of TiNiV, interdendritic eutectic enriched in Ni, and particles enriched in titanium of the Ti_2_Ni type with a size of 1–2.5 µm (Figure 6c). A characteristic feature of the Ti_2_Ni phases is their regular round shape.

Quantitative microanalysis data show that fine particles of TiNiV and B2 phase, which also includes vanadium, are formed in alloy No. 1 (Table 1). 

The addition of 1% vanadium to the alloy leads to the formation of a structure formed in the form of enlarged columnar dendrites (Figure 6a), eutectic (Figure 6b) and fine particles of TiNiV. It has been established that particles of the Ti_2_Ni type in the alloy under study are uniformly distributed over the volume and are formed during the peritectic reaction. The average size of dendritic cells in alloys of this composition is 9 μm.

The microstructure of alloy No. 2, obtained on optical and scanning electron microscopes, is shown in Figure 7. Adding 2 at.% V leads to the formation of a structure with areas of dendritic crystallization and coarse particles ranging in size from 7 to 15 µm (Figure 7a,b). An analysis of the state diagram of the Ti–Ni binary system allows to make an assumption that during the crystallization of alloy No. 2, the formation of primary peritectic dendrites occurs. The matrix interacts with the melt according to the peritectic mechanism without the formation of a eutectic. The average grain size is 23 µm. 

According to quantitative microanalysis data (Table 2), the structure of the alloy includes: a matrix of TiNi grains; dendrites enriched in Ti with V content; dark particles of arbitrary shape enriched in titanium of the Ti_2_Ni(V) type with a size of 7 to 15 μm, located singly and in clusters throughout the volume of the alloy; fine particles of TiNiV, crystallized separately in the matrix; particles of the Ti_4_Ni_2_O(V) type; segregation phase enriched in titanium TiNi(V) (Figure 7c). According to the results of microanalysis, it was found that vanadium is present in all the listed structural components of alloy No. 2. Due to the presence of vacancies in the lattice of the TiNi phase with the B2 structure, O dissolve in it, which can lead to the formation of oxycarbonitrides with a composition of the type Ti_4_Ni_2_(O). Oxygen in this compound has the ability to stabilize triple phases [22]. Ti_4_Ni_2_O oxides also containing vanadium have a lamellar, round or regular rhombic shape (Figure 7c). 

**Figure 8:**
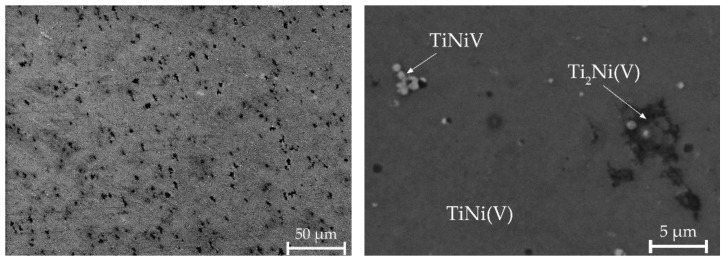
Microstructure of alloy No. 3.

A different morphology without areas with a dendritic structure is observed in alloy No. 3. It has been established that the alloy contains regions with the Ti_2_Ni(V) phase, uniformly distributed TiNiV particles, and a TiNi(V) solid solution (Figure 8, Table 3). Quantitative microanalysis data show a higher content of vanadium atoms observed in the B2 phase.

Quantitative microanalysis data show that fine particles of TiNiV and B2 phase, which also includes vanadium, are formed in alloys. This agrees with the data obtained from the results of X-ray diffraction analysis, from which it can be seen that the introduction of vanadium leads to an increase in the lattice parameter of the R-phase. 

**Figure 9:**
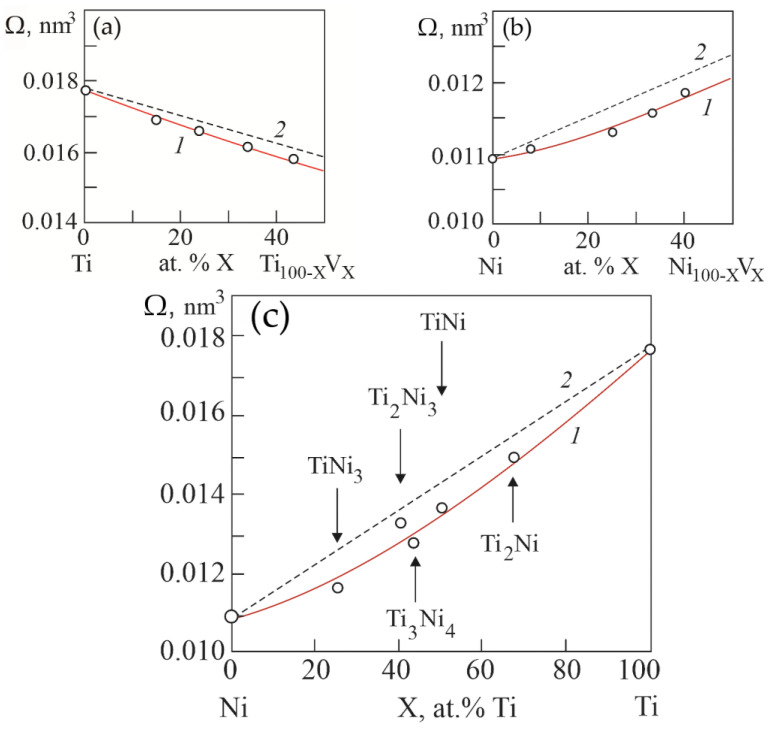
Concentration dependences of atomic volumes in binary systems Ti–V (**a**), Ni–V (**b**) and Ni–Ti (**c**). Curves 1 were obtained from the concentration dependences of the lattice parameters in the considered systems. Curves 2 are theoretical dependences calculated according to Zen’s law.

Figure 9 shows the concentration dependences of atomic volumes Ω in binary systems Ti–V, Ni–V and Ni–Ti, obtained on the basis of calculations based on the concentration dependences of lattice parameters in the considered systems. It can be seen that in binary systems Ti–V, Ni–V and Ni–Ti there is a negative deviation of atomic volumes from Zen’s law. That is, there is a “compression” of the crystal lattice in solid solutions and compounds in the considered binary systems.

The energy of a crystal having a metallic bond can be written as:

(1) $$U=-\frac{Ae^{2}}{\Omega^{\frac{1}{3}}}+\frac{B}{\Omega^{\frac{2}{3}}}+\frac{Ce^{2}}{\Omega }$$

Here, *A*, *B*, *C* are constants; e is the electron charge. According to Equation (1), a negative deviation from Zen’s law of atomic volume on concentration dependences leads to an increase in the energy of the crystal lattice in solid solutions in binary systems Ti–V, Ni–V and in compounds of the Ni–Ti system.

**Figure 10:**
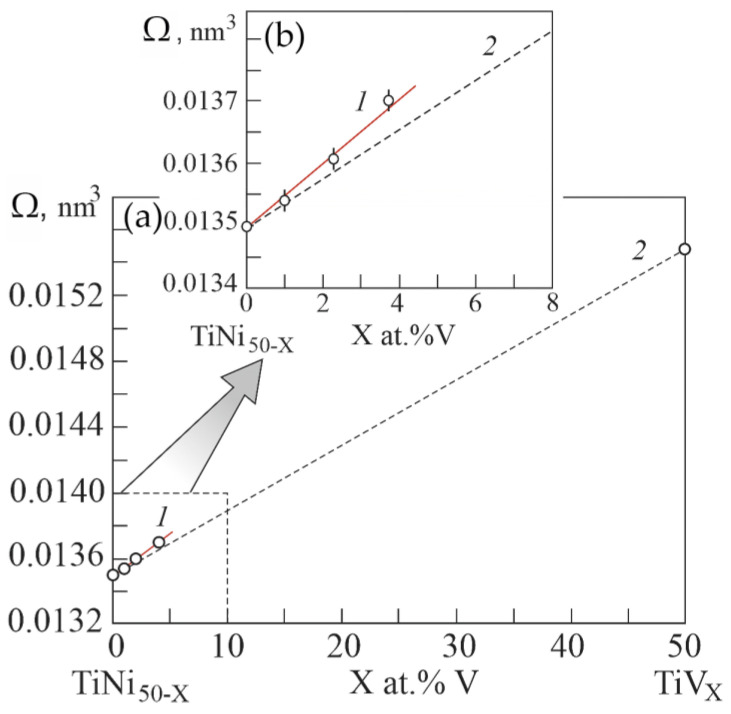
Concentration dependence of atomic volumes in the ternary system Ti_50_Ni_50−X_V_X_ (**a**). Curve 1 was obtained from the data from the experiment of the concentration dependence of lattice parameters in the Ti_50_Ni_50−X_V_X_ system. Curve 2 is theoretical dependence, calculated according to Zen’s law. Inset (**b**) shows an enlarged scale of the concentration dependence of atomic volumes in the region of low concentrations V.

Figure 10 shows the experimental atomic volumes versus the concentration of atoms V in the B2 phase based on the EDS results. It can be seen that doping with the third component V of the TiNi binary compound leads to a positive deviation of the atomic volume on the concentration dependence of Zen’s law. Whereas in binary systems Ti–V and Ni-V doping with V leads to a negative deviation of the concentration dependence of the atomic volume from Zen’s law. According to Equation (1), the positive deviation of the atomic volume on the concentration dependence from Zen’s law reflects a decrease in the energy of the crystal lattice in the TiNi-based alloy upon alloying with V. Such a decrease in the energy of the crystal lattice in the phase with the B2 structure possibly contributes to the phase transition to the R phase. Thus, doping with V atoms of TiNi compounds leads to an increase in their volume.

### 3.3. Multiple Shape Memory Effect of Ti-Ni-V System Alloys

**Figure 11:**
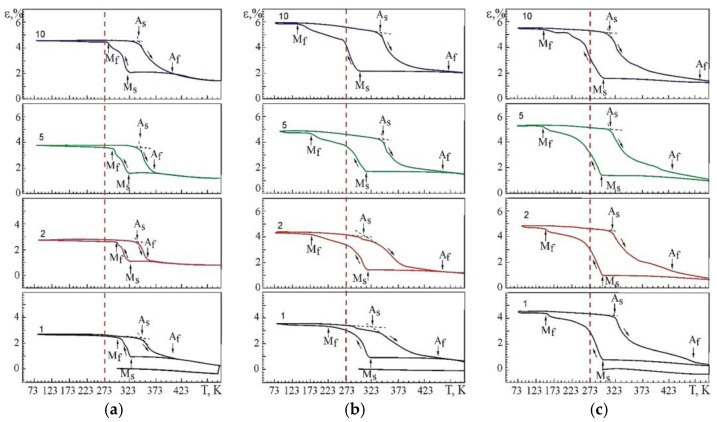
Multiple SME in alloys: (**a**) No. 1; (**b**) No. 2; (**c**) No. 3. The numbers indicate the numbers of cycles after thermal cycling through the MT region.

The results of studying the multiple SME of the Ti–Ni–V system alloys by the macrodeformation method in tensile conditions with a constant load are shown in Figure 11 and in Table 4. Curves in Figure 11 demonstrate a similar character of the change in deformation during multiple SME in the investigated alloys. Change of ε = f(T) during multiple SME depending on the concentration of atoms V is due to the two-stage behavior of the B2→R→B19’ MT occurring in the studying alloys. 

Figure 11 shows the temperature dependences of the change in deformation under constant load during thermal cycling. It should be noted that the temperature dependences of deformation in the alloys under study at multiple SMEs show the evolution of these dependences both with an increase in the thermal cycle number and with a change in the concentration of atoms V. The experimental dependences ε(T) at multiple SME in alloy No.1 demonstrate the minimum values of the accumulated strain both during cooling and heating in relation to alloys with a high content of V.

Studies of the microstructure showed that alloys doped with vanadium are heterogeneous in structure and chemical composition, the main share of inhomogeneities is associated with the presence of fine TiNiV particles in the matrix, which are an obstacle to strain accumulation, reducing the amount of B2 matrix material responsible for MT. 

**Figure 12:**
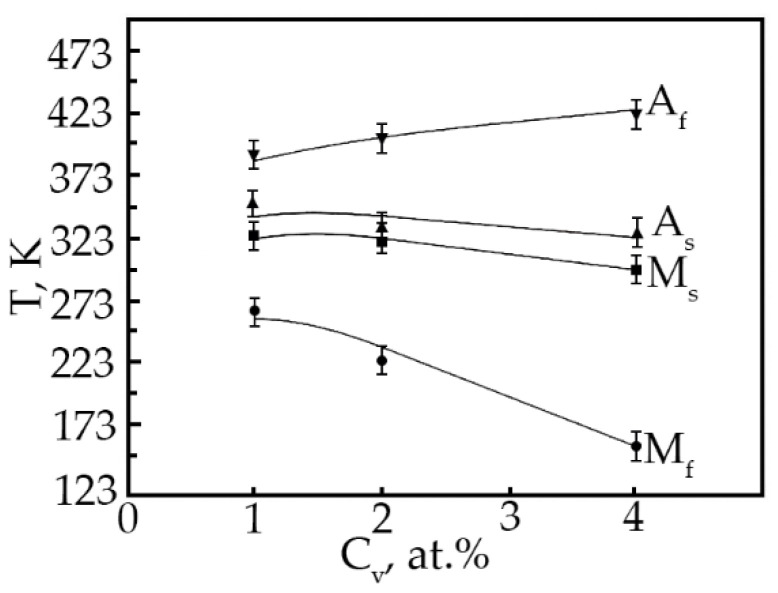
Concentration dependence of the characteristic MT temperatures in alloys doped with V.

Based on the analysis of the ε(T) dependences, the characteristic MT temperatures, and the concentration dependences of the temperatures of the onset of the transition in TiNi alloys doped with vanadium were determined (Figure 12). The temperatures M_S_, M_f_ and A_S_ with increasing concentration V, shift to the region of low temperatures, and the temperatures M_S_, A_S_ decrease insignificantly (~by 25 K), while the temperature M_f_ drops from 300 to 153 K. Such a character of the change in the characteristic temperatures with an increase in the concentration of V atoms in TiNi-based alloys clearly shows that the MT under load leads to a broadening of the interval. Probably, this is caused by structural inhomogeneities—these are TiNiV particles, which act as an obstacle to the onset of MT, while Ti_2_Ni(V) and Ti_4_Ni_2_O(V) particles make an additional contribution to the expansion of the MT temperature range.

**Figure 13:**
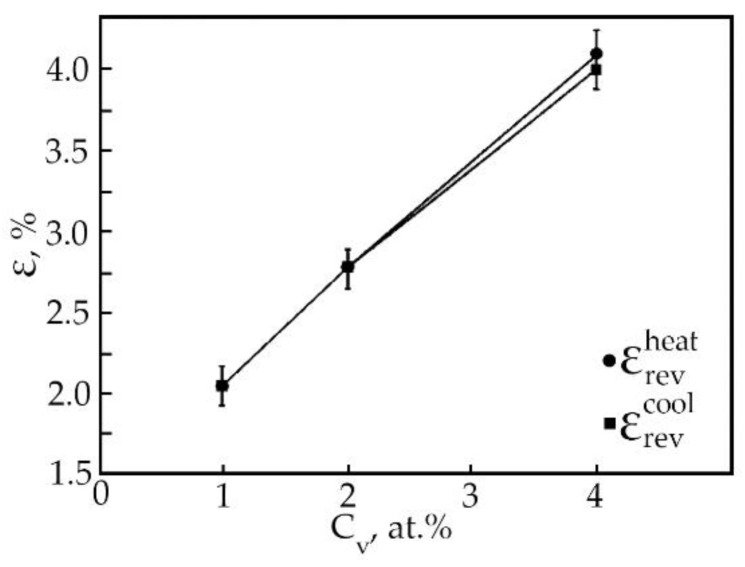
Concentration dependences of reversible deformation (●—during heating, ■—during cooling) of alloys doped with V.

**Figure 14:**
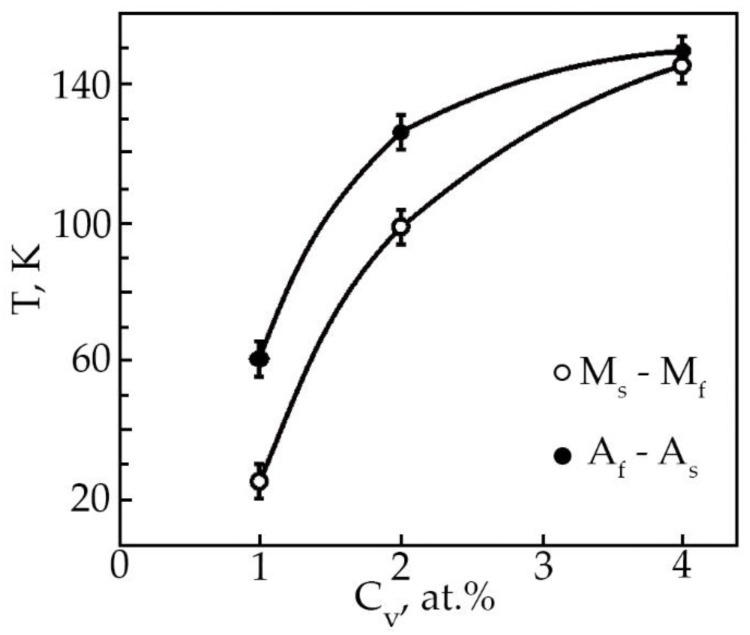
Concentration dependences of the width of the temperature intervals of forming TiNi-based alloys doped with V.

The results of processing the dependences ε(T) of deformation at multiple SMEs made it possible to construct the concentration dependences of the reversible deformation of alloys based on TiNi doped with vanadium (Figure 13). It has been found that the reversible strain increases linearly with an increase in the vanadium concentration. Doping of TiNi-based alloys with vanadium leads to an increase in the width of the temperature intervals of direct MT upon cooling of the alloy under load with increasing concentration V (Figure 14). When alloys No. 1, 2, and 3 are heated under load, the transition temperature range weakly depends on the concentration of atoms V. An important point is that, depending on the concentration of atoms V, there is a different character of the ratio between the temperature intervals of direct and reverse MT. This phenomenon is clearly illustrated in Table 5. In the first two cases, when (M_S_ − M_f_) < (A_f_ − A_S_) in alloys No. 1 and 2 during MT under load, martensitic transformation in these alloys under load with decreasing temperature leads to the formation of defects and their complexes during the movement of interfacial borders. Then, during reverse MT under load, these defects begin to impede the movement of interfacial boundaries.

This phenomenon leads to a broadening of the temperature range of the reverse MT under load. In alloy No. 3, the temperature intervals of direct and reverse MT coincide (M_s_ − M_f_)~(A_f_ − A_s_). This means that the defects and stress fields between the new MT plates that have arisen as a result of the movement of interfacial boundaries during the growth of martensite plates in the austenite phase have exactly the same effect on the movement of interfacial boundaries during the reverse transformation.

In all alloys, after heating, the residual strain is insignificant. In an alloy based on TiNi doped with vanadium instead of nickel, with a concentration of 4 at.%, the value of residual strain is 0.1% (Figure 13). The appearance of residual strain during multiple SME is associated with the accumulation in the process of deformation as plastic strain, which is the main mechanism of dissipation of elastic energy during MT. Defects that arise during plastic deformation led to the destruction of the long-range order in intermetallic compounds. These phenomena have a significant effect on the mobility of interfaces and lead to irreversible effects [23].

**Figure 15:**
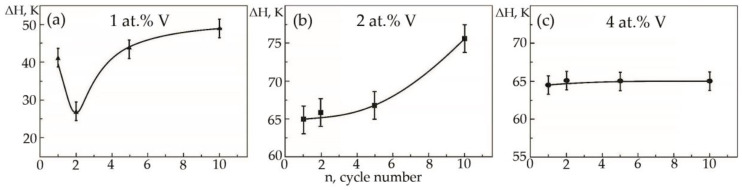
Width of the hysteresis loop on the temperature dependence of deformation under constant load on the cycle number in alloys: (**a**)—No. 1; (**b**)—No. 2; (**c**)—No. 3.

Dependences of the hysteresis loop width on the temperature dependence of deformation under constant load on the cycle number in alloys based on titanium nickelide with various vanadium concentrations were plotted (Figure 15). In this case, the width of the temperature hysteresis ΔH was measured over the widest possible place on the dependence of the accumulated strain during cooling and heating (the middle of the hysteresis).

**Figure 16:**
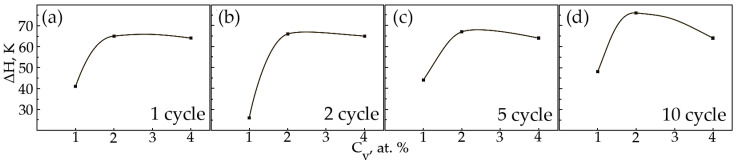
The width of the hysteresis loop on the temperature dependence of the deformation under constant load on the concentration V: (**a**)—1 cycle; (**b**)—2nd cycle; (**c**)—5th cycle; (**d**)—10 cycle.

In alloys with a concentration of 1 and 2 at.% V, an increase in the width of the hysteresis loop is observed during thermal cycling (Figure 15a,b). As for the sharp fall of the width of the hysteresis loop of sample No 1, the second cycle always proceeds differently from the first cycle. The difference lies in the fact that during martensitic transformation under the action of a load, the paths of the interface boundary during loading and unloading on the first cycle and on the second cycle do not coincide, but starting from the third and further, it passes exactly along its route. Apparently, obstacles in the form of particles of secondary phases in a smaller amount than in the first cycle were encountered on the way to the movement of the interface in the second cycle. In an alloy with 4 at.%, vanadium ΔH does not change significantly, with an increase in the number of thermal cycles (Figure 15c). It has been established that with increasing concentration V, the width of the hysteresis loop ΔH exhibits a similar character of dependences on thermal cycling (Figure 16). An increase in the width of the MT hysteresis loop during phase hardening also occurs due to an increase in the density of defects with an increase in the number of thermal cycles and the resulting long-range displacement fields that begin to interact with each other under load reaching saturation by the 10th cycle. The defects formed during direct martensitic transformation under load cause phase hardening, which leads to a noticeable deceleration of the motion of interfacial boundaries during reverse MT under load.

In contrast to the above two alloys, in alloy No. 3, a completely different phenomenon is observed: thermal cycling does not change the width of the hysteresis loop under load (Figure 9c). The authors suggest that in the alloy with direct MT under load, after the first cycle, a fairly stable complex of crystal structure defects is formed, which then weakly depends on subsequent thermal cycles through the MT area.

An experimental study of the structural-phase states and the multiple SME of alloys based on titanium nickelide alloyed with V showed that, despite an increase in the volume fraction of TiNiV particles and secondary phases based on Ti_2_Ni(V), with an increase in the vanadium content, an increase in the accumulated strain during the multiple SME is observed, as during cooling, and during heating, which indicates the prospect of using alloys of the Ti–Ni–V system as actuating elements in technology.

## 4. Conclusions

An experimental study of the phase composition, structure, crystal structure, shape memory parameters of TiNi-based alloys doped with 1, 2 and 4 at.% V has been carried out. The research results were summarized in the form of the following conclusions:

1. All investigated alloys at room temperature contain a multiphase mixture consisting of intermetallic compounds with compositions: TiNi (B2, R and B19′) and Ti_2_Ni. It has been established that at room temperature, the volume fraction of the B19′ phase decreases with increasing concentration of V atoms. This trend in the change in the structural-phase composition indicates that doping of TiNi alloys with vanadium atoms leads to an increase in the stability of the high-temperature B2 and rhombohedral R-phase. An increase in the atomic volume with an increase in the concentration of the doping element V was established.
2. The microstructure of TiNi alloys doped with vanadium is characterized by the presence of finely dispersed TiNiV particles in the TiNi(V) matrix, which are not an obstacle to the accumulation of deformation during multiple SME.
3. Doping with vanadium of alloys of the Ti–Ni–V system leads to an increase in the temperature interval for the manifestation of the multiple SME.
4. It has been established that the value of the reversible deformation of the multiple SME both during heating and during cooling increases linearly with an increase in the vanadium concentration.
5. Titanium nickelide alloy doped with 4 at.% vanadium is promising for use as actuating elements in promising technology.

## Figures and Tables

**Table 1:** Quantitative elemental composition of the structural components of alloy No. 1.

Structural Component	Composition, at.%		
	Ti (±0.2)	Ni (±0.1)	V (±0.1)
TiNi(V) matrix	49.85	48.88	1.27
Ti_2_Ni particles	60.38	39.62	-
TiNiV particles	33.27	32.87	33.86
TiNi eutectic	40.5	59.95	-

**Table 2:** Quantitative elemental composition of the structural components of alloy No. 2.

Structural Component	Composition, at.%			
	Ti (±0.2)	Ni (±0.1)	V (±0.1)	O (±0.2)
TiNi(V) matrix	47.66	50.06	2.28	-
TiNi(V) dendrites	58.64	40.26	1.1	-
Ti_2_Ni(V) particles	63.43	35.88	0.69	-
TiNiV particles	36.43	33.25	30.32	-
Ti_4_Ni_2_O(V) particles	59.16	30.5	1.23	9.11
Segregation phase TiNi(V)	51.81	45.77	0.24	-

**Table 3:** Quantitative elemental composition of the structural components of alloy No. 3.

Structural Component	Composition, at.%		
	Ti (±0.2)	Ni (±0.1)	V (±0.1)
TiNi(V) matrix	47.17	49.56	3.27
Ti_2_Ni(V) particles	65.65	33.52	0.83
TiNiV particles	36.41	31.33	32.26

**Table 4:** Characteristics of multiple SME in TiNi alloys doped with V.

SME Parameters	Content V, at.%		
	1	2	4
M_S_ (K)	325 ± 5	323 ± 5	298 ± 5
M_f_ (K)	300 ± 5	224 ± 5	153 ± 5
A_S_ (K)	345 ± 5	324 ± 5	322 ± 5
A_f_ (K)	405 ± 5	450 ± 5	470 ± 5
ε_rev_^cool^ (%) *	2.1 ± 0.1	2.8 ± 0.1	4.1 ± 0.1
ε_rev_^heat^ (%) *	2.1 ± 0.1	2.8 ± 0.1	4.0 ± 0.1
ε_resid_ (%) *	-	-	0.1
ΔH (K)	41 ± 5	65 ± 5	64 ± 5

ε_rev_^cool^ *—reversible deformation on cooling; ε_rev_^heat^ *—reversible deformation on heating; ε_resid_ *—residual strain.

**Table 5:** Relations between MT intervals and the width of hysteresis loops from thermal cycling (where 1, 5, 10 is the number of cycle).

Alloy	Relations between the Intervals of Direct and Reverse MT	Relationships between Hysteresis Loop Widths from Thermal Cycling	M_S_ − M_f_, K	∆H_1_, K	∆H_5_, K	∆H_10_, K
1	(M_s_ − M_f_) < (A_f_ − A_s_)	∆H1 < ∆H5 < ∆H10	25 ± 5	41 ± 5	44 ± 5	48 ± 5
2	(M_s_ − M_f_) < (A_f_ − A_s_)	∆H1 < ∆H5 < ∆H10	99 ± 5	65 ± 5	67 ± 5	76 ± 5
3	(M_s_ − M_f_)~(A_f_ − A_s_)	∆H1~∆H5~∆H10	145 ± 5	64 ± 5	65 ± 5	65 ± 5
